# Transoesophageal echocardiography-guided balloon-assisted percutaneous closure of a large secundum atrial septal defect in a pregnant woman: a case report

**DOI:** 10.1093/ehjcr/ytae014

**Published:** 2024-01-06

**Authors:** Radityo Prakoso, Rina Ariani, Brian Mendel, Oktavia Lilyasari

**Affiliations:** Division of Pediatric Cardiology and Congenital Heart Disease, Department of Cardiology and Vascular Medicine, National Cardiovascular Centre Harapan Kita, Universitas Indonesia, Letjen S. Parman St No.Kav. 87, Slipi, Palmerah, West Jakarta City, 11420 Jakarta, Indonesia; Division of Non-invasive Diagnostic and Cardiovascular Imaging, Department of Cardiology and Vascular Medicine, National Cardiovascular Centre Harapan Kita, Universitas Indonesia, Letjen S. Parman St No.Kav. 87, Slipi, Palmerah, West Jakarta City, 11420 Jakarta, Indonesia; Division of Pediatric Cardiology and Congenital Heart Disease, Department of Cardiology and Vascular Medicine, National Cardiovascular Centre Harapan Kita, Universitas Indonesia, Letjen S. Parman St No.Kav. 87, Slipi, Palmerah, West Jakarta City, 11420 Jakarta, Indonesia; Department of Cardiology and Vascular Medicine, Sultan Sulaiman Government Hospital, Serdang Bedagai, Negara 58 No. 315, Firdaus, Sei Rampah, Serdang Bedagai City, 20995 North Sumatera, Indonesia; Division of Pediatric Cardiology and Congenital Heart Disease, Department of Cardiology and Vascular Medicine, National Cardiovascular Centre Harapan Kita, Universitas Indonesia, Letjen S. Parman St No.Kav. 87, Slipi, Palmerah, West Jakarta City, 11420 Jakarta, Indonesia

**Keywords:** Atrial septal defect, Balloon-assisted, Pregnant, Case report, Non-fluoroscopy, Occluder

## Abstract

**Background:**

According to the 2018 European Society of Cardiology guidelines, atrial septal defect (ASD) closure can be performed during pregnancy but is rarely indicated. In this case, we demonstrate the viability of percutaneous balloon-assisted ASD closure without fluoroscopy in a pregnant woman.

**Case summary:**

A 23-year-old G_3_P_2_A_0_ woman who was 20 weeks pregnant had primary complaints of breathlessness [New York Heart Association functional class (NYHA fc) III and IV] for 1 week prior to admission. A transthoracic echocardiography showed a dilatation of the right atrium (RA), a dilated right ventricle, a dilated main pulmonary artery (28.1 mm), and an oval-shaped 22 × 33 mm-sized secundum ASD with a left-to-right shunt. Despite optimal pharmacological treatment, the NYHA fc persisted. Under transoesophageal echocardiography monitoring, we introduced a 40 mm Cera™ ASD Occluder (Lifetech, China) via the delivery sheath. The device was deployed in the usual position; however, despite numerous placement adjustments, the left atrium disc kept getting dislodged to the RA and could not engage correctly. Therefore, we decided to use a balloon-assisted approach using a sizing balloon of No. 34 mm. The device was successfully positioned, and a wiggle test was conducted to make sure that the device remained stable. The patient was able to give birth to the child normally several months later.

**Discussion:**

Despite the fact that pregnant women with ASD receive a very low dose of radiation, it is nevertheless recommended to avoid radiation because this demographic is particularly vulnerable to it. It is possible to treat a large ASD in pregnant women with a successful balloon-assisted approach.

**Learning points**

- To understand that a balloon-assisted procedure could be used on a pregnant woman with a large atrial septal defect (ASD).
- To understand that percutaneous non-fluoroscopy guidance for ASD closure is feasible for pregnant women.

## Introduction

Atrial septal defect (ASD) is one of the most common cardiovascular conditions encountered during pregnancy. In pregnant ASD patients with severe and haemodynamically significant defects, arrhythmias and increased dyspnoea can develop, especially during the second and third trimesters. In such conditions, ASD closure should be performed even during pregnancy when pharmacological treatment is unable to ameliorate the symptoms.^1–3^ According to the 2018 European Society of Cardiology (ESC) guidelines, ASD closure can be performed during pregnancy but is rarely indicated.^4^ Nevertheless, serious safety concerns are raised by percutaneous device closure during pregnancy regarding radiation dose exposure to the mother and fetus.^1,5,6^ In this case report, we demonstrate the viability of percutaneous balloon-assisted ASD closure without fluoroscopy in a pregnant woman.

## Summary figure

**Figure:**
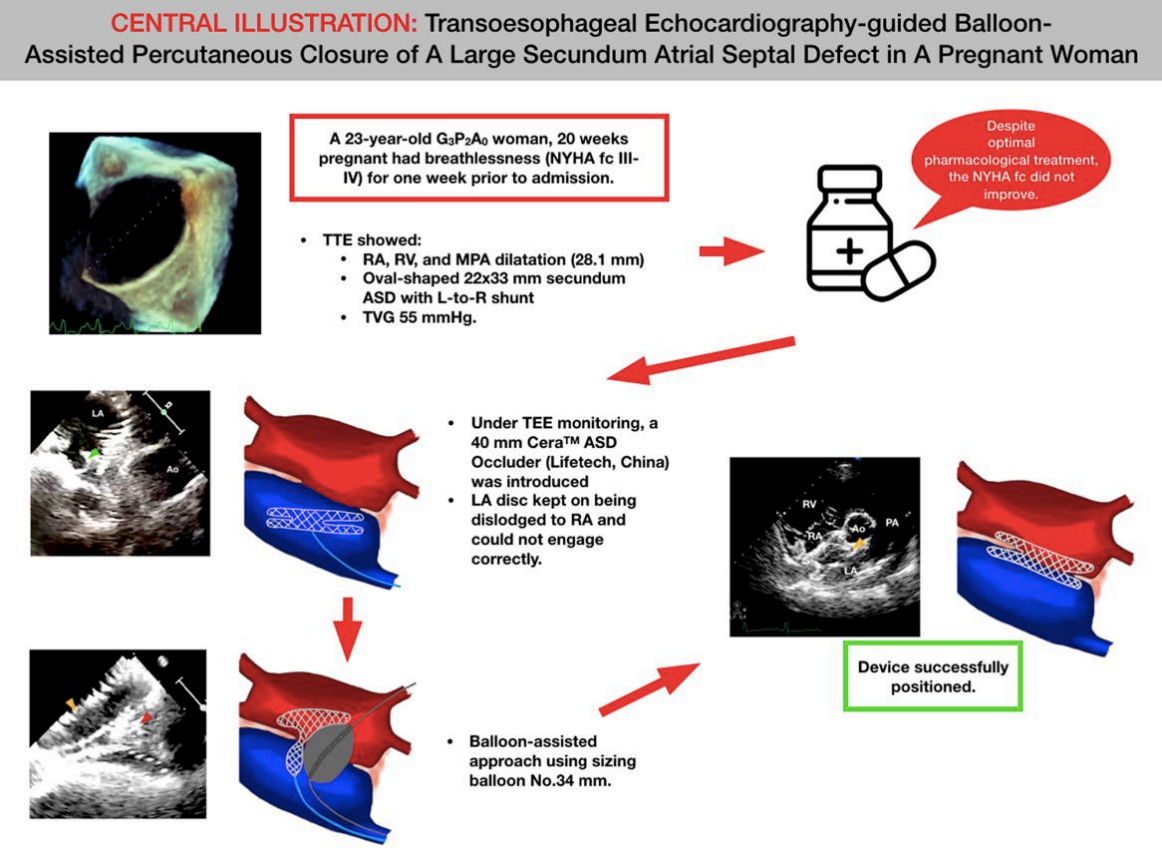


## Case summary

**Figure 1:**
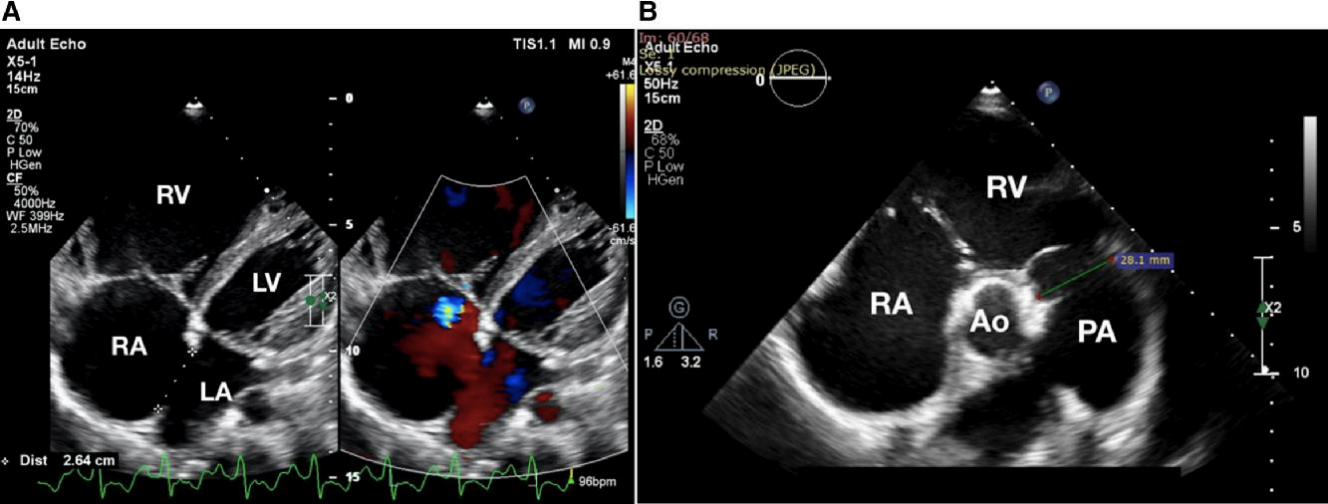
Echocardiography features in pregnant atrial septal defect patients. A transthoracic echocardiography showed a dilatation of the right atrium, right ventricle, main pulmonary artery (28.1 mm), and an oval-shaped 22 × 33 mm-sized secundum atrial septal defect, with an L-to-R shunt. (*A*) A four-chamber view. (*B*) A parasternal short-axis view. Ao, aorta; LA, left atrium; LV, left ventricle; RA, right atrium; RV, right ventricle.

**Figure 2:**
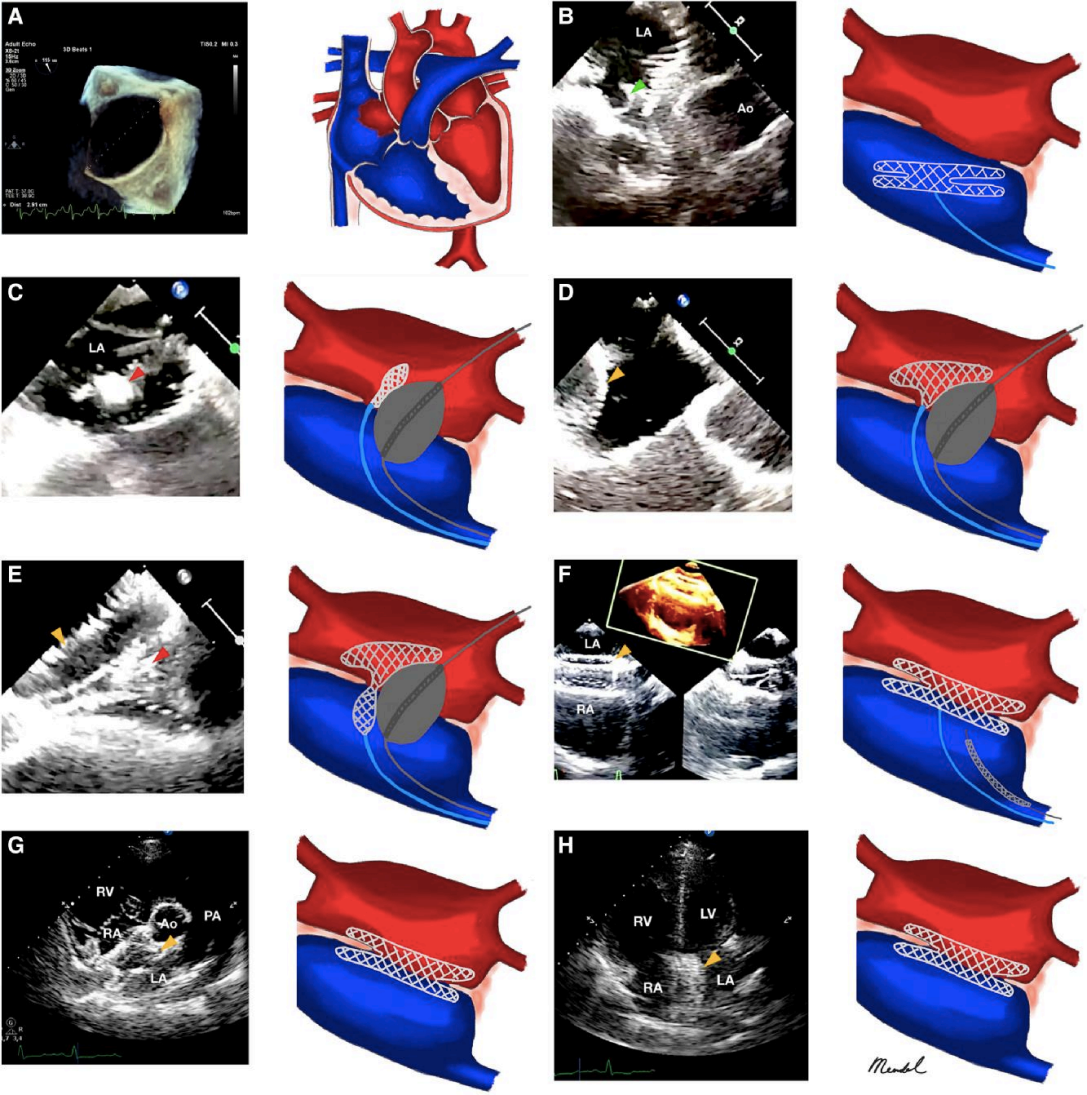
Balloon-assisted non-fluoroscopy secundum atrial septal defect closure in pregnant women. (*A*) An oval-shaped 22 × 33 mm-sized secundum atrial septal defect with a left-to-right (L-to-R) shunt and transvalvular gradient of the tricuspid valve of 55 mmHg. (*B*) A transoesophageal echocardiography short-axis image indicates that the left atrial disc dislodged from the right atrium and could not engage correctly (*green arrowhead*). (*C*–*E*) A balloon-assisted approach using a sizing balloon of No. 34 mm (*red arrowhead*). After the device was finally stowed, a wiggle test was conducted to make sure that it remained stable (*orange arrowhead*), which was confirmed in a transoesophageal echocardiography bicaval view, (*F*) transthoracic echocardiography parasternal long-axis view (*G*), and apical four-chambered view (*H*).

A 23-year-old G_3_P_2_A_0_ woman who was 20 weeks pregnant had primary complaints of breathlessness [New York Heart Association functional class (NYHA fc) III and IV] for 1 week prior to admission. Her past medical history was not available. Her vital signs showed a blood pressure of 119/69 mmHg, heart rate of 90 b.p.m., respiratory rate of 18 times/min, and SpO_2_ of 99%. She had a body mass index of 16.6 kg/m^2^. There was no palpable lower extremities oedema. During auscultation, there was a typical S1 with a wide and fixed split S2. Her electrocardiogram showed a sinus rhythm with right axis deviation, P pulmonale, a PR interval of 0.18 s, a QRSd of 0.12 s, and right ventricular hypertrophy. A transthoracic echocardiography showed a dilatation of the right atrium (RA), a dilated right ventricle, a dilated main pulmonary artery (28.1 mm; see *Figure 1*; see Supplementary material online, *Video S1*), and an oval-shaped 22 × 33 mm-sized secundum ASD with a left-to-right (L-to-R) shunt and transvalvular gradient (TVG) of the tricuspid valve of 55 mmHg (*Figure 2A*; see Supplementary material online, *Video S2A*).

Despite optimal medical treatment with sildenafil 25 mg thrice daily (t.i.d.), bisoprolol fumarate 1.125 mg once daily (o.d.), furosemide 40 mg twice daily (b.i.d.), and spironolactone 25 mg o.d., the NYHA fc persisted. By a decision of the surgical conference, percutaneous ASD closure without fluoroscopy was planned on this patient. Prior to the procedure, we took a bedside echocardiogram using the segmental sequential analysis method to rule out any further conditions such as anomalous pulmonary venous return. During the procedure, the patient was intubated and put under general anaesthesia. A puncture of the right femoral vein was done, and a multipurpose 6 Fr catheter and heparin 5000 IU intra-sheath were introduced. With only a midoesophageal short-axis view, transoesophageal echocardiography (TEE) guidance, and pressure tracking, the multipurpose catheter was inserted from the inferior vena cava to the RA and crossed towards the left atrium (LA). Right heart catheterization (RHC) was not done prior to percutaneous ASD closure in our centre if the shunt was still a L-to-R one with no evidence of pulmonary vascular disease. The Amplatz Super Stiff™ guidewire (Boston Scientific, USA) was introduced and positioned in the left upper pulmonary vein (LUPV). By comparing the midoesophageal short-axis view with the midoesophageal bicaval view, the 14 Fr delivery sheath was inserted using the ‘over-the-wire’ technique towards the LUPV. The Amplatz Super Stiff™ guidewire (Boston Scientific) was then removed. Under TEE monitoring, we introduced a 40 mm Cera™ ASD Occluder (Lifetech, China) via the delivery sheath. The device was deployed in the usual position; however, despite numerous placement adjustments, the LA disc kept getting dislodged to the RA and could not engage correctly (*Figure 2B*). Therefore, we decided to use a balloon-assisted approach using a sizing balloon of No. 34 mm (*Figure 2C–E*). Fundamentally, the midoesophageal bicaval and short-axis views allowed for safe execution of the balloon-assisted approach. The two ‘rails’ are supposed to pass through the left and right femoral veins, respectively. The balloon sizing was inserted from the left femoral vein and secured in the LUPV. With TEE guidance, the balloon was then positioned into the ASD and inflated until it completely occluded the ASD. In order to ensure that the device fitted in well, it was deployed while the balloon was progressively deflated. The balloon and the wire were then carefully retracted after the balloon had been deflated. After the device was finally stowed, a wiggle test was conducted to make sure that the device remained stable (*Figure 2F–H*; Supplementary material online, *Video S2B* and *C*).

The patient was discharged 2 days later after her symptoms had subsided. She received an additional 80 mg aspirin o.d. Sildenafil was stopped since her TVG went down from 55 to 26 mmHg. During follow-up in the ward and cardiology clinic 1 day, 1 month, 6 months, and 1 year post-procedure, it was found that the patient and the baby did not have any issues.

## Discussion

### Eliminating radiation exposure to pregnant women and fetuses during percutaneous procedures

Despite having given the patient the maximum recommended dose of pharmacotherapy, the symptoms continued to worsen, and therefore, we opted to conduct percutaneous closure while the patient was still pregnant rather than waiting until the baby was delivered, even though the saturation rate was still 99%.

Percutaneous device closure during pregnancy raises serious safety concerns regarding radiation dose exposure to the mother and the fetus.^1,5^ The potential of percutaneous ASD closure without fluoroscopy was initially performed by Ewert *et al*.^7^ Recently, many hospitals, including ours, have treated both newborns and adults using non-fluoroscopy–guided closure. Our institution has demonstrated a high success rate that is comparable with the use of conventional fluoroscopy.^8–10^ This method is particularly beneficial to special populations, notably to young children and expectant mothers who are more susceptible to radiation exposure, making it the appropriate treatment for these populations.^7^

### The approach of large atrial septal defect percutaneous closure in pregnant women

A large ASD is not always defined in the same way. However, ASDs that require devices wider than 25 mm are typically thought of as large. Arrhythmias and increasing dyspnoea may appear during pregnancy in people with severe and haemodynamically significant defects. Arrhythmias (2%), as well as thrombo-embolic consequences (5%), are maternal concerns associated with an ASD. When severe pulmonary hypertension is present, there is a very significant risk of maternal (up to 50%) and fetal (up to 60%) mortality.^1,5,10^ Invasive RHC is advised in accordance with the ESC 2018 guidelines if there is diagnostic uncertainty and to support significant therapeutic decisions.^4^ However, no RHC procedure was performed prior to ASD closure in our patient.

When a patient has a large ASD, the LA disc frequently refuses to align with the plane of the inter-atrial septum (IAS) and herniates into the RA, as it did in our patient. Additionally, our procedure was rendered more difficult due to the large ASD oval morphology. A small LA size, unstable rims, a floppy inferior rim, and an irregular LA curvature—either singularly or collectively—lead to incorrect device deployment. Surgery can be performed to fix large ASD defects, but there is a substantial risk of maternal and fetal morbidity and mortality.^5,11^ We, therefore, made the decision to carry out the closure percutaneously.

Predictable delivery is made possible in patients with a large ASD, thanks to the balloon-assisted approach. To align the LA disc with the IAS without slipping through the defect, a sizing balloon is used in this procedure. In specific instances of an oval-shaped ASD, balloon inflation may change the defect’s shape to match the balloon’s somewhat circular shape. The sole distinction between the fluoroscopy and the non-fluoroscopy approaches is that the former allowed only the operator to view a part of the procedure, while the latter provided a complete picture to everyone present. However, this method may be one of the best options for reducing radiation exposure in pregnant women.

### Limitation

No RHC procedure was performed prior to ASD closure in this patient.

## Conclusion

Despite the fact that pregnant women with ASD receive a very low dose of radiation, it is nevertheless recommended to avoid radiation because this demographic is particularly vulnerable to it. It is possible to treat a large ASD in pregnant women with a successful balloon-assisted approach.

## Lead author biography


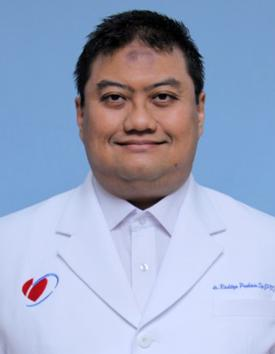
Radityo Prakoso, MD, FACC, FESC, FSCAI, is a paediatric interventional cardiologist from the National Cardiovascular Centre Harapan Kita, Jakarta, Indonesia. He is renowned for his contribution in zero fluoroscopy septal defects and ductus arteriosus closure in Indonesia. Currently, he is the president of the Indonesian Heart Association. He has contributed in numerous books and international publications, mainly in the fields of paediatric cardiology and congenital heart disease. He is also invited as a guest speaker and panellist in national and international cardiology-related conferences or congresses.

## Supplementary material

Supplementary material is available at *European Heart Journal – Case Reports* online.

## Supplementary Material

ytae014_Supplementary_Data

## Data Availability

The data that support the findings of this study are available from the authors upon reasonable request.
